# Old and New Aspects of *H. pylori*-Associated Inflammation and Gastric Cancer

**DOI:** 10.3390/children9071083

**Published:** 2022-07-20

**Authors:** Andreea Ligia Dincă, Lorena Elena Meliț, Cristina Oana Mărginean

**Affiliations:** Department of Pediatrics I, George Emil Palade University of Medicine, Pharmacy, Science and Technology of Târgu Mureș, Gheorghe Marinescu Street No 38, 540136 Târgu Mureș, Romania; dinca.andreea87@yahoo.ro (A.L.D.); marginean.oana@gmail.com (C.O.M.)

**Keywords:** *Helicobacter pylori*, inflammation, cytokines

## Abstract

*H. pylori* is involved in the development of 80% of gastric cancers and 5.5% of all malignant conditions worldwide. Its persistence within the host’s stomach causes chronic inflammation, which is a well-known hallmark of carcinogenesis. A wide range of cytokines was reported to be involved in the initiation and long-term persistence of this local and systemic inflammation. IL-8 was among the first cytokines described to be increased in patients with *H. pylori* infection. Although, this cytokine was initially identified to exert a chemoattracting effect that represents a trigger for the activation of inflammatory cells within *H.*-*pylori*-infected mucosa, more recent studies failed in encountering any association between IL-8 and *H. pylori* infection. IL-6 is a multifunctional, pleiotropic and multipotent cytokine involved in mediating the interaction between innate and adaptive immunity with a dichotomous role acting as both a proinflammatory and an anti-inflammatory cytokine depending on the signaling pathway. IL-1α functions as a promoter of angiogenesis and vascular endothelial cell proliferation in gastric carcinoma since it is closely related to *H.*-*pylori*-induced inflammation in children. IL-1β is an essential trigger and enhancer of inflammation. The association between a low IL-1β level and an increased TNF-α level might be considered a risk factor for peptic ulcer disease in the setting of *H. pylori* infection. IL-10 downregulates both cytotoxic inflammatory responses and cell-mediated immune responses. *H. pylori* uses the immunosuppressive role of IL-10 to favor its escape from the host’s immune system. TGFβ is a continuous inflammatory mediator that promotes the adherence of *H. pylori* to the host’s cells and their subsequent colonization. The role of *H.*-*pylori*-induced inflammatory responses in the onset of gastric carcinogenesis seems to represent the missing puzzle piece for designing effective preventive and therapeutic strategies in patients with *H.*-*pylori*-associated gastric cancer.

## 1. Introduction

*Helicobacter pylori* (*H. pylori*), which is a Gram-negative, microaerophilic bacterium that is known to affect over 50% of the worldwide population; it occurs initially during childhood, but if left untreated, might persist for life, resulting in a wide spectrum of gastropathies, such as chronic gastritis, peptic ulcer, gastric adenocarcinoma or gastric-associated lymphoid tissue lymphoma [1,2]. Thus, the most important long-term life-threatening complication of this infection is related to gastric carcinogenesis, which is reported to be involved in the development of 80% of all gastric cancers and 5.5% of all malignant conditions worldwide [3,4]. *H. pylori* persistence within the host’s stomach causes chronic inflammation, which is a well-known hallmark of carcinogenesis. 

*H. pylori* infection is particularly important in pediatric patients, mainly due to its high prevalence in this age group. A very recent meta-analysis noted that the prevalence of this infection is still very high in children and adolescents [5]. The most important risk factors identified in this meta-analysis for acquiring this infection in pediatric subjects were: lower economic status, more siblings or children, having a mother infected with *H. pylori*, having a sibling or siblings infected with *H pylori*, room sharing, no access to a sewage system, drinking unboiled or non-treated water, and older age. 

*H.*-*pylori*-associated inflammation depends on both bacterial virulence factors and the host’s immune responses. Several *H. pylori* pathogenicity features were shown to contribute to the development of this inflammatory process, such as cag-pathogenicity island (cagPAI), vacuolating cytotoxin A, or pathogen-associated molecular patterns (PAMPs) comprising lipopolysaccharides and flagellin [6]. In terms of the host’s immune responses, both innate and adaptive immunity play crucial roles in the activation of the immune cells’ receptors and subsequent synthesis and secretion of a wide spectrum of proinflammatory cytokines [7]. *H. pylori* was shown to trigger both a local inflammation, which is not surprising, but also a low-grade systemic inflammation. Therefore, once it colonizes the gastric mucosa, *H. pylori* has the ability to trigger a local inflammation by attracting neutrophils and lymphocytes at this level, which further results in the release of several chemotactic proteins in the stomach [8]. According to the Sydney Gastritis Classification System, the predominance of neutrophils characterizes acute inflammation of the gastric mucosa, while lymphocyte and plasmocyte predominance is specific for chronic gastritis [9]. The previously mentioned systemic subclinical inflammatory status associated with *H. pylori* infection seems to be involved in the development of several extraintestinal conditions, such as cardiovascular disorders, glaucoma, anemia, stroke, rosacea, eczema, chronic hives, thrombocytopenic idiopathic purpura, diabetes, thyroid disease or Alzheimer’s disease [10]. In addition, increased *H. pylori* antibodies were found to be associated with coronary artery disease [11], high systolic blood pressure and arterial stiffness in diabetic patients [12]. The relationship between *H. pylori* infection and hematological disorders was suggested by several recent studies that indicated that patients with idiopathic thrombocytopenic purpura should benefit from screening tests for *H. pylori* infection independent of the presence of gastrointestinal symptoms [13,14,15,16]. Moreover, the successful eradication of this bacterium resulted in a subsequent increase in platelet count [17]. Iron-refractory iron deficiency anemia is another peculiar condition encountered especially in children with *H. pylori* infection in whom the efficacy of iron supplementation considerably improved after successful eradication of this infection, but the reported results remain controversial [18]. 

The assessment of *H.*-*pylori*-associated low-grade inflammatory status might represent an important step toward carcinogenesis prevention by taking into account the fact that this inflammation represents the cornerstone of carcinogenesis. Multiple studies assessed the usefulness of several laboratory parameters in detecting the inflammation associated with this infection, which included the complete cellular blood count parameters (leukocytes, erythrocytes, neutrophils, lymphocytes, platelets, red blood cell volume distribution width or medium platelets volume), C-reactive protein (CRP), erythrocyte sedimentation rate, neutrophils/lymphocytes ratio or platelets/lymphocytes ratio [19,20]. Moreover, certain parameters were used for gastric cancer screening [20]. The involvement of complete cellular blood parameters in the development of gastric cancer was suggested by several studies that emphasized the role of platelets in the secretion of pro-inflammatory cytokines [21], as well as the role of lymphocytes as a potentially protective factor against tumor growth [22]. It was shown that *H. pylori* infection is associated with an increase in both neutrophils and platelets, as well as with a decrease in lymphocyte count [23,24], once more strengthening the strong relationship between *H. pylori* and gastric cancer. 

*H. pylori* infection remains a challenging infection in pediatrics, and the controversies related to the diagnosis, treatment, mechanisms of resistance to antibiotics and especially its involvement in gastric carcinogenesis require a more thorough approach.

*The aim of this review was to assess the insights regarding the relationship between H. pylori-associated inflammation and gastric cancer.*

## 2. The Trialogue between *H. pylori*, Cytokines and Gastric Cancer

The negative impact of *H. pylori* on a host’s homeostasis depends on the bacterial adhesion and invasion of the gastric epithelium. The close interaction between *H. pylori* and the epithelial surface is enabled by the outer membrane proteins known as adhesins, which are divided into Helicobacter outer membrane proteins (Hop) and Hop-related (Hor) subgroups. The Hop group comprises: the blood group antigen-binding adhesions (BabA), which favor the adherence of this bacterium to Lewis^b^ antigens and ABO blood group antigen; sialic acid binding adhesion protein (SabA), which enhances the adherence to already damaged gastric epithelial cells via an increased expression of the receptor sialyl-dimeric-Lewis^x^ glycosphingolipid; *AlpA* and *AlpB* genes also contribute to cellular adhesion and modulation of proinflammatory intracellular signaling cascades; the *HopZ* gene, which is an adhesion protein located at the *H. pylori* bacterial surface; and the outer inflammatory protein A (OipA), which is a strong trigger of inflammation via IL-8 production [25]. The effect on the gastric epithelium is strengthened by several *H. pylori* virulence factors or bacterial products translocated into the host gastric epithelial cells through a type 4 secretion system (T4SS) encoded in the cag pathogenicity island (cagPAI) since it was shown that only 20% of the colonizing bacteria are in close contact with the epithelium [25,26]. Aside from CagA, which is the effector protein encoded at one end of cagPAI, probably the most virulent *H. pylori* factor and a true risk factor for peptic ulcer disease and gastric cancer, other bacterial cell wall components are also translocated via the T4SS, such as muropeptides or peptidoglycan, with the latter one being recognized by NOD1, which is an intracellular PAMP recognition receptor leading to the upregulation of proinflammatory immune responses via nuclear factor NF-κB activation [27]. Along with cagPAI, other virulence factors were found to be directly involved in damaging the host epithelium, among which were the vacuole-inducing toxin (VacA); urease, which acts as both an enzyme for buffering the pH in the gastric microenvironment and as an adhesion factor; and high-temperature requirement A product, which controls the serine protease activity acting on the adhesion molecule E-cadherin [25,28,29]. All the previously mentioned mechanisms eventually contribute to the disruption of epithelial cell barriers and subsequent cell apoptosis. 

### 2.1. Cytokines Secretion

Cytokine secretion represents the core of the inflammatory process caused by *H. pylori*. Thus, *H. pylori* has the ability to induce the secretion of a wide spectrum of cytokines, most of which are secreted by gastric epithelial cells that are implicated in the development of *H.*-*pylori*-associated gastropathies. 

#### 2.1.1. Interleukin (IL)-8

It is a well-documented fact that *H. pylori* infection is associated with an increase in IL-8 levels, both in vitro and in vivo [30], where it was among the first cytokines described to be produced by infected gastric epithelium [30,31,32,33]. As we already mentioned, IL-8 production is related to the presence of CagA in infecting *H. pylori* strains, where it is associated with the recruitment of neutrophils [25,31]. After its secretion, IL-8 exerts a chemoattracting effect and triggers the activation of inflammatory cells within *H.*-*pylori*-infected mucosa [34]. Therefore, gastric epithelial cells represent a true reservoir of IL-8 [35,36] and once *H. pylori* colonizes the gastric mucosa, it will primarily activate the recruitment and activation of neutrophils at this level [37]. In the setting of prolonged *H. pylori* infection, the long-term production of IL-8 leads to the recruitment of leukocytes in the gastric mucosa, representing a major step in the regulation of immune-inflammatory responses [38]. IL-8 can be activated via various signaling pathways, such as NF-κB, inducible transcription factors and activator protein-1 (AP-1), but the greatest effect is displayed by NF-κB [39,40]. 

It was also suggested that the activity of IL-8 depends on its gene polymorphism, where it was shown that the A allele of this gene, located on chromosome 4, is associated with an increased IL-8 production that amplifies the inflammatory response [41,42]. Moreover, it was recently noted that the presence of this allele at position -251 of the IL-8 gene might be associated with the host’s increased susceptibility toward developing *H. pylori* infection and its subsequent persistence based on the significant association between this allele and *H. pylori* infection in the studied group [43]. A recent study pointed out that gender might play a crucial role in influencing the severity of gastric lesions, revealing that the IL-8 251 AT gene polymorphism has a protective effect for acute chronic gastritis development in women and represents a risk factor in males [44]. Nevertheless, the results reported in the literature remain controversial since the meta-analysis of Xue et al. revealed the contrary, concluding that the IL-8-251AA genotype is not associated with *H. pylori* infection, but it might increase the risk of developing gastric cancer [45]. Similarly, a study performed on healthy populations in Indonesia failed to identify a significant association between IL-8 gene polymorphisms and *H. pylori* infection [46] (Table 1). 

*H.*-*pylori*-derived outer membrane vesicles were recently shown to increase the IL-8 mRNA expression and IL-8 secretion [47]. In addition, these vesicles have the ability to also activate the NF-κB pathway, indicating that the effect of *H. pylori* on gastric epithelial cells is not entirely related to the bacterium itself. The magnitude of IL-8 stimulation and expression seems to be related to the interaction between H.-pylori-derived outer membrane vesicles and different gastric epithelial cell lines depending on their different origin and susceptibility to PAMPs [47]. Surprisingly, a very recent study found a negative correlation between *H. pylori* nodular gastritis and the expression of IL-8 mRNA, but at the same time, pointed out that *H.*-*pylori*-positive patients tend to have an increased expression of IL-8 as compared with non-infected subjects [48]. The authors explained this negative correlation based on the increased bacterial load in the corpus of *H. pylori* positive nodular gastritis patients, which might result in a lower recognition mediated by *H. pylori* immune escaping mechanisms in these patients (Table 1). 

Although IL-8 was initially considered to have a key role in triggering immune-inflammatory responses as a result of *H. pylori* infection, further studies questioned this hypothesis, revealing that the effect of this cytokine might vary and depends on both the host’s immune response and bacterial peculiarities. These findings were recently supported by Chen et al., who found no correlation between IL-8 serum levels and hepcidin levels, which is a well-known regulator hormone of iron absorption, in children with *H. pylori* infection [49], suggesting once more that the role of this interleukin might not be the same as described initially. 

#### 2.1.2. IL-6

IL-6, whose gene is located on chromosome 7, is a multifunctional, pleiotropic and multipotent cytokine expressed by multiple human cells, such as monocytes, lymphocytes and macrophages, as well as endothelial and intestinal epithelial cells [50]. It functions as an essential mediator of both immunity and inflammation [50,51]. One of the most important roles of IL-6 consists of the stimulation of acute-phase reactants through the interaction with IL-1β for the synthesis of CRP [52]. Based on the relationship between its serum levels and coronary heart disease events [53,54,55], several studies noted a direct association between serum IL-6 and the risk or severity of coronary heart disease [53,54,55,56,57]. Unsurprisingly, further studies raised the concern regarding whether *H. pylori* is connected to this pathology based on the alterations caused in the inflammatory and immune responses [58]. The results on this topic remain controversial since several studies revealed a positive association [59,60,61,62], while others failed in identifying a significant association [63,64,65] (Table 1). 

Aside from this important role, IL-6 is also involved in the regulation of proliferation and differentiation of B and T lymphocytes, favoring the production of IFN-γ production in T cells, stimulating B-cell differentiation as a response to antibody-forming plasma cells, enabling the secretion of immunoglobulins in activated B lymphocytes and eventually in triggering several carcinogenesis-related processes, such as proliferation, migration and angiogenesis [51]. Therefore, IL-6 might be defined as a mediator between innate and adaptive immunity. Beyond the implications of IL-6 in immune responses, this cytokine also functions as an endocrine and metabolic regulator [66] (Table 1). 

In terms of *H. pylori* infection, it is well documented that this infection is associated with higher levels of IL-6 independent of the studied tissue or sample. In terms of blood samples, several studies pointed out a significant positive association between serum IL-6 levels and *H. pylori* antibodies [58,67]. These findings are also supported by studies performed on gastric biopsy samples, which indicated that IL-6 gastric mucosal levels are increased in *H.*-*pylori*-positive gastritis and they diminish after the successful eradication of this infection [41]. Unfortunately, the gastric levels of this cytokine remain high in *H.*-*pylori*-positive gastric carcinoma [68]. Nevertheless, a more recent study that assessed IL-6 polymorphisms in *H.*-*pylori*-positive gastric biopsy samples found no correlation between the IL-6 174 polymorphism and the risk of gastric carcinoma [43] (Table 1). 

Two signaling pathways contribute to the exertion of IL-6 functions, resulting in a dichotomous role of this cytokine acting as both a pro-inflammatory cytokine via trans-signaling mode and as an anti-inflammatory one via the classic signaling pathway [69]. Thus, IL-6 binds to its receptor (IL-6Rα) and coreceptor (IL-6Rβ), resulting in activation of the JAK/STAT signaling pathway, with the signals being transduced via gp130, which is a strong inducer of STAT3 [70]. This mechanism is responsible for abnormal immune responses representing the cornerstone for the progression and invasion of gastric carcinoma [70,71]. Moreover, once the carcinogenesis is initiated, the process is maintained due to the persistent activation of this signaling, along with TNF-α–NF-κB, resulting in the subsequent activation of intrinsic cytokine signaling for promoting gastric cancer cell growth [50]. Except for carcinogenesis, *H. pylori* might also be involved in iron deficiency since it is well documented that IL-6 is a crucial player in the activation of hepcidin transcription via STAT3 signaling during inflammation [72], which represents a potential explanation for *H.*-*pylori*-induced iron deficiency [73]. Thus, Jing et al. recently concluded that IL-6 might represent a useful screening tool for the detection of early gastric cancer [72]. Similarly, a recent study performed on pediatric patients with *H. pylori* noted a strong association between IL-6 and increased hepcidin levels [49]. Another study that assessed *H.*-*pylori*-positive children indicated that innate immune players, such as toll-like receptor 2 (TLR) and TLR4, play key roles in the expression of IL-6 in *H.*-*pylori*-infected children [74,75] (Table 1). 

IL-6 is definitely a key player in inflammatory and immune responses triggered by *H. pylori*, where it is a true mediator between innate and adaptive immune responses, but its further roles in gastric carcinogenesis and other *H.*-*pylori*-related disorders remain to be elucidated. 

#### 2.1.3. IL-1

IL-1 is a cytokine that is strongly related to carcinogenesis, where it was shown to be produced by several types of malignant tumor cells and it plays multiple roles in the growth of solid tumors. Nevertheless, it seems that its role depends on the tumoral tissue, where it was found that IL-1 promotes the growth of gastric carcinoma but it hinders the growth of breast, melanoma and ovarian carcinomas [50]. The IL-1 family comprises 11 members: IL-1α and IL-1β, which exert similar roles and have the same receptor, but also IL-18, IL-33, IL-36α, IL-36, IL-36γ, IL-1R antagonist, IL-36Ra, IL-37 and IL-38 [76,77]. Out of these, the most important ones in terms of *H. pylori* infection are IL-1α, IL-1β and IL-18. 

##### IL-1α

IL-1α is expressed by activated macrophages and malignant gastric cells, where they are able to induce autocrine growth factor, endothelial growth factor and endothelial growth factor receptor. Moreover, it was noted that it functions as a promoter of angiogenesis and vascular endothelial cell proliferation in gastric carcinoma [50]. Anti-IL-1α or anti-IL-1 receptor antagonist might represent a novel targeted therapy in gastric cancer since it was shown that it might suppress malignant cell growth in a gastric cancer cell line [50]. This endpoint might be explained by the fact that patients with *H.*-*pylori*-induced gastritis, especially children, were found to have an overexpression of this cytokine in their gastric mucosa, which is strongly correlated with *H.*-*pylori*-associated inflammation. These findings are sustained by the same authors who highlighted that *H. pylori* infection enhances the expression of IL-1α via the trans-signaling pathway IL-6/IL-6 soluble receptor in the setting of chronic gastritis [78]. Therefore, IL-1α is a potent pro-inflammatory cytokine that exhibits a strong effect during both inflammation and carcinogenesis (Table 1). 

##### IL-1β

IL-1β can also be defined as an important trigger and enhancer of inflammation since it is produced by multiple types of cells, such as immune cells, epithelial cells and fibroblasts [79]. Thus, we might state that the host’s inflammatory response is closely regulated by IL-1β since it was shown that it augments this response to both endogenous and exogenous stimuli, also inducing the expression of other cytokines, such as IL-2, IL-6, IL-12 and TNF-α, but also of other pro-inflammatory mediators, among which are inducible nitric oxide synthetase and cyclooxygenase 2 [77,80]. The complex interrelationship between these cytokines, *H. pylori* infection and IL-1β was emphasized by a study performed on acute coronary syndrome patients, where it was shown that the coexistence of *H. pylori*-seropositivity and certain IL-1β polymorphisms is associated with higher levels of high-sensitivity CRP, resulting in an increased risk of ST-segment elevation myocardial infarction [81]. In addition, genetic variants in the promoter regions of the IL-1β gene were recently shown to alter cytokine expression, creating a hypoacidity environment involved in enabling the long-term persistence and colonization of *H. pylori* [82]. Nevertheless, the influence of IL-1β gene polymorphisms on *H. pylori* infection might be closely related to ethnicity or geographical area [83]. The genetic variants of this cytokine seem to also influence the success of *H. pylori* eradication regimens in patients with functional dyspepsia [84]. 

IL-1β was found to be strongly related to the innate immune system based on the fact that the activation of two key components of innate immune responses, namely, toll-like receptor (TLR) 2 and TLR 4, were indicated to play an essential role in the expression of this cytokine in *H.*-*pylori*-infected pediatric patients [85]. Nevertheless, a recent study pointed out that IL-1β might be involved in the pathogenesis of peptic ulcers regardless of the presence of *H. pylori* infection [86]. Moreover, the authors concluded that the association between a decrease in the IL-1β level, along with an increase in TNF-α, might be considered a risk factor for peptic ulcer disease in the setting of *H. pylori* infection. Another study that assessed *H.*-*pylori*-positive patients with and without nodular gastritis pointed out that IL-1β was increased only in the antrum of patients without nodular gastritis, suggesting that the expression of this cytokine might be related to the bacterial load [48] (Table 1). 

In terms of gastric cancer, IL-1β, which is strongly involved in the activation of NF-κB, has the ability to stimulate the proliferation of malignant gastric tumor cells through a tyrosine kinase pathway, which is associated with granulocyte-macrophage colony-stimulating factor [50]. In addition, this cytokine seems to be associated with CRP levels and lower survival rates in patients with gastric carcinoma [87], indicating that it definitely triggers a strong pro-inflammatory response in patients with *H. pylori* with long-term life-threatening risks. Moreover, these findings demonstrate that IL-1β acts synergistically, with IL-6 substantially augmenting the inflammatory responses triggered by *H. pylori.* Another important interrelation with a great impact on gastric carcinogenesis was identified between IL-1β 511 T and IL-8 251 T gene polymorphisms, which in the setting of increased virulence of *H. pylori* results in a higher risk of gastric cancer [44] (Table 1). Thus, the upregulation and increased secretion of IL-1β might be considered a major causative factor for the hyperproliferation of gastric epithelial cells and subsequent oncogenesis [88]. 

#### 2.1.4. IL-18

Our knowledge of the role of IL-18 in gastric carcinogenesis is incontestable due to its strong correlations with tumor progression and metastasis that lead to angiogenesis based on its influence on the production of proangiogenic factor and thrombospondin in gastric cells, which further expresses the IL-18 receptor through the JNK pathway [50]. Furthermore, it amplifies gastric cancer cells and migration and it has the ability to activate the NF-κB pathway, therefore interacting with other cytokines that have the same effect [50]. IL-18 serum levels might be considered a prognostic factor for patients with gastric cancer [50], where it was highlighted that patients with this type of tumor and increased IL-18 serum levels present a lower survival rate when compared with patients with the same condition but lower levels of circulating IL-18 (Table 1).

#### 2.1.5. IL-10

IL-10 is a well-known immunosuppressive cytokine that is able to downregulate both cytotoxic inflammatory responses and cell-mediated immune responses [89]. IL-10 is encoded by a protein located on chromosome 1, and two single nucleotide polymorphisms in the promoter region were associated with low IL-10 production: a C-A base transition at position -592 and a C-T base transition at position -819 [43]. As a result of these transitions, decreased IL-10 production leads to severe gastric inflammation and a consequently higher risk of developing gastric carcinoma in *H.*-*pylori*-positive patients [90]. Thus, *H. pylori* has the capacity to upregulate IL-10 as an escape mechanism for enabling its survival and persistence through effective suppression of the immune response [91]. Nevertheless, the suppressive effects of IL-10 on inflammation activation were recently emphasized in a study performed on *H.*-*pylori*-infected human cells [92]. Although the results reported in the literature are controversial, a recently published study showed that the IL-10 gene is biologically important in patients diagnosed with *H. pylori* infection [43] (Table 1). 

IL-10 acts similarly to IL-6 via the activation of STAT3 based on the initial activation of tyrosine phosphorylation of tyk2 and Jak1, which subsequently phosphorylate the IL-10 receptor intracellular domain [50]. The implications of IL-10 in immune responses are explained by its capacity to regulate the function of antigen-presenting cells, as well as both the synthesis and expression of macrophages and the pro-inflammatory cytokines produced by the T-cells [93]. In terms of gastric cancer, the level of circulating IL-10 was shown to be directly dependent on the gastric cancer stage, therefore influencing the prognosis of these patients since it stimulates angiogenesis and suppresses immune responses that enable tumor progression [50] (Table 1). 

The immunosuppressive role of IL-10 is widely used by *H. pylori* in order to escape the host’s defense mechanisms and to enable its survival and long-term persistence within the gastric microenvironment. 

#### 2.1.6. TNF-α

TNF-α is an endotoxin-induced glycoprotein belonging to a larger family of cytokines that act as mediators of systemic inflammation and acute phase reactions. As an endogenous pyrogen and the main regulator of immune cells, this cytokine has the ability to induce fever, apoptosis, inflammation and cachexia [94,95]. TNF-α is mainly involved in mediating the acute inflammatory responses to Gram-negative bacteria, along with other infectious agents, where it is responsible for a wide spectrum of complications. Therefore, this cytokine mediates the inflammatory responses during infection with *H. pylori* as a result of the stimulating effect of *H. pylori* lipopolysaccharide on macrophages, leading to the synthesis of a great amount of TNF-α [96]. Furthermore, TNF-α acts as both a recruitment and an activator factor for monocytes, macrophages and neutrophils by stimulating their migration to the infection site [97]. It has been long known that TNF-α is significantly higher in patients with *H. pylori* infection than in healthy controls, as well as in those with histologically demonstrated gastritis and peptic ulcers, suggesting that it might contribute to the gastric injuries caused by this infection [98]. Recent findings also verified this statement [96]. The expression of TNF-α is correlated with the bacterial load according to Mansilla-Vivar et al., who pointed out that this cytokine is significantly higher in *H.*-*pylori*-positive patients without nodular gastritis, which is a well-known sign of increased bacterial load, than in those with nodular gastritis caused by *H. pylori* infection [48]. In addition, a recent study revealed a strong positive association between *H. pylori* IgG antibodies and the serum concentrations of TNF-α [86]. The authors also suggested that this cytokine might be an indicator of the severity of tissue damage in patients with peptic ulcers. The involvement of TNF-α in the etiopathogenesis of peptic ulcers might be explained by its chemotactic effect on T and B cells and the consecutive progression to peptic ulcers [99]. Aside from its implications in peptic ulcers and gastric cancer, TNF-α seems to also play a role in the etiology of other human disorders, such as Alzheimer’s disease, major depression, non-gastric cancers or inflammatory bowel disease [86] (Table 1). 

The role of TNF-α in carcinogenesis was shown by several previous researchers, who indicated that it has a double-edged role: as an upregulator of the nitric-oxide-dependent pathway responsible for the inhibition of DNA repair and the promotion of DNA damage, and as an inductor of angiogenesis [50]. In addition, this cytokine also impacts the NF-κB pathway, resulting in a stimulation of other cytokines, matrix metalloproteinases and angiogenic factors, with great implications for tumor cell survival and metastasis [50]. The NF-κB pathway represents a bridge between *H. pylori* infection and gastric carcinogenesis that is sustained by a complex array of cytokines, including TNF-α. Moreover, *H. pylori* has the ability to secrete a protein meant to stimulate TNF-α production, the TNF-α-inducing protein (Tipα), which will enable *H. pylori* to act as a tumor promoter [100]. This protein is a contributor to *H.*-*pylori*-induced gastric carcinogenesis via a pathway that differs from those of CagA and VacA. Nevertheless, the results in terms of carcinogenesis remain controversial since not all of the studies published in the past demonstrated an increase of this cytokine in patients with gastric cancer [101] (Table 1). 

Based on all the aforementioned data, TNF-α represents a core cytokine in the complex array of cytokines involved in *H.*-*pylori*-triggered inflammation. 

#### 2.1.7. Transforming Growth Factor (TGF) β

TGF β is a multifunctional cytokine that was first described in the early 1980s [102]. The TGF β family consists of activins, inhibins, growth differentiation factors, bone morphogenetic proteins, TGF β isoforms and glial-cell-derived factors [103]. Three isoforms of this cytokine were described: TGF β1, which is expressed by hematopoietic, epithelial and endothelial cells; TGF β2, which is expressed by epithelial and neuronal cells; and TGF β3, which is mainly expressed in mesenchymal cells [104]. TGF β might act via two signaling pathways: SMAD and non-SMAD, with both resulting in the regulation of cell proliferation and differentiation [105]. The SMAD signaling pathway is responsible for the regulation of target genes expression, such as epithelial-mesenchymal transcription (EMT) factors snail family zinc finger, zinc finger E-box binding homeobox 1 [106], plasminogen activator inhibitor 1 [107], matrix metalloproteinases [104], IL-6 and connective tissue growth factor [108]. The non-SMAD signaling pathway involves the activation of c-Jun N-terminal kinase, the external signal-regulated kinase and the p38 mitogen-activated protein kinase signaling pathways [109]. Therefore, TGF β might be defined as a continuous inflammatory mediator and it can be induced by several cell types, including macrophages, foam cells and lymphocytes (Table 1). 

Several previous studies reported that TGF β promotes the adherence of *H. pylori* to the host’s cells and their subsequent colonization [110], suggesting that its increased expression might be an indicator of the severity of *H.*-*pylori*-related non-metaplastic atrophic gastritis [111]. It was also suggested that the expression of this cytokine depends on the *H. pylori* virulence, where it was shown that the inhibition of T-cell proliferation and immune responses due to *H. pylori* VacA results in an increased adherence of *H. pylori* to the gastric epithelial cells via the upregulation of TGF β expression [112]. Similar findings indicated that serum TGF β, along with IL-17A and IL23, are significantly increased in patients with *H.*-*pylori*-positive gastritis and peptic ulcers as compared with *H.*-*pylori*-negative subjects [113]. 

Altered TGF β signaling pathways seem to be involved in gastric cancer development. Both TGF β1 and TGF β2 were found to be significantly increased in early and advanced gastric carcinoma when compared with control samples [114], suggesting their role as predictors of poor prognosis in patients with this type of malignant condition. In addition, TGF β1 was shown to promote the invasion and metastasis of gastric cancer [105] (Table 1). Moreover, increased TGF β1 serum levels were found in *H.*-*pylori*-positive gastric cancer patients [115]. It is well-documented that the TGF β1 promoter is methylated in the setting of gastric cancer and the level of methylation was reported to be significantly higher in gastric mucosa infected with *H. pylori* versus uninfected gastric mucosa [116]. The SMAD-induced EMT enhances the invasion and metastasis of *H.*-*pylori*-positive gastric cancer. Furthermore, the subsequent emergence of cancer stem cells resulting in a loss of polarity and cell–cell adhesion in epithelial cells eventually leads to a disruption in the basal membrane and extraintestinal metastasizing [117,118]. Aside from EMT signaling, the signaling of SMAD-dependent bone morphogenic proteins was also involved in the complex evolution of *H. pylori* infection toward gastric carcinogenesis, with a documented role in intestinal metaplasia and gastric cancer, with both being associated with *H. pylori* infection [119,120] (Table 1).

Undoubtedly, TGF β is strongly related to *H. pylori* infection progression and the subsequent progression toward gastric cancer. 

**Table 1:** The role of cytokines in inflammation and *H. pylori* infection.

Cytokine	Secretion	Role	Observations
**IL-8**	Production is related to the presence of CagA in infecting *H. Pylori* strains → associated with the recruitment of neutrophils [25,31] Activation of inflammatory cells within *H.*-*pylori*-infected mucosa [34]	Recruitment of leukocytes in the gastric mucosa → the regulation of immune-inflammatory responses [38] Allele -251 of the IL-8 gene is associated with the host’s increased susceptibility to developing *H. pylori* infection [43] IL-8 251 AT gene polymorphism → a protective effect for acute chronic gastritis development in women and a risk factor in males [44] Xue et al → IL-8-251AA genotype is not associated with *H. pylori* infection, but it increases the risk of developing gastric cancer [45]	Gastric epithelial cells → reservoir of IL-8 [35,36] IL-8 can be activated via various signaling pathways, such as NF-κB [39,40]. *H.*-*pylori*-derived outer membrane vesicles → increase the IL-8 mRNA expression and IL-8 secretion [47] A negative correlation between *H. pylori* nodular gastritis and the expression of IL-8 mRNA, showing that *H.*-*pylori*-positive patients have an increased expression of IL-8 [48]
**IL-6**	Expressed by multiple human cells, such as monocytes, lymphocytes, macrophages, and endothelial and intestinal epithelial cells [50]	Essential mediator of both immunity and inflammation [50,51] Stimulation of acute-phase reactants [52] A direct association between serum IL-6 levels and the risk or severity of coronary heart disease [53,54,55,56,57] The regulation of proliferation and differentiation of B and T lymphocytes, favoring the production of IFN-γ production in T cells, enabling the secretion of immunoglobulins in activated B lymphocytes and triggering several carcinogenesis-related processes [51] IL-6 acts as a pro-inflammatory cytokine and as an anti-inflammatory [69] Involved in carcinogenesis [70,71] Crucial player in the activation of hepcidin transcription via STAT3 signaling during inflammation [72]	A mediator between innate and adaptive immunity → this cytokine also functions as an endocrine and metabolic regulator [66] Association between serum IL-6 levels and *H. pylori* antibodies [58,67] No correlation between the IL-6 174 polymorphism and the risk of gastric carcinoma [43] TLRs 2 and 4 play key roles in the expression of IL-6 in *H.*-*pylori*-infected children [74,75]
**IL-1α**	Expressed by activated macrophages and malignant gastric cells [50]	Functions as a promoter of angiogenesis and vascular endothelial cell proliferation in gastric carcinoma [50] Is able to induce autocrine growth factor endothelial growth factor and endothelial growth factor receptor [50] A pro-inflammatory cytokine → strong effect during both inflammation and carcinogenesis [50]	Children with *H.*-*pylori*-induced gastritis → an overexpression of this cytokine in their gastric mucosa → correlated with *H.*-*pylori*-associated inflammation [50] Anti-IL-1α or anti-IL-1 receptor antagonist → novel targeted therapy in gastric cancer [50]
**IL-1β**	Produced by immune cells, epithelial cells and fibroblasts [79]	Important trigger and enhancer of inflammation [79] Induces the expression of other cytokines, such as IL-2, IL-6, IL-12 and TNF-α, but also nitric oxide synthetase and cyclooxygenase 2 [77,80] Related to the innate immune system ➔ activates TLR2 and TLR 4 Coexistence of *H. pylori* seropositivity and certain IL-1β polymorphisms are associated with higher levels of high-sensitivity CRP ➔ increased risk of ST-segment elevation myocardial infarction [81] Genetic variants in the promoter regions of the IL-1β gene → alter cytokine expression → a hypoacidity environment involved in enabling long-term persistence and colonization of *H. pylori* [82] IL-1β gene polymorphisms influence *H. pylori* infection and might be closely related to ethnicity or geographical area [83] The genetic variants of this cytokine → influence the success of *H. pylori* eradication regimens in patients with functional dyspepsia [84] Involved in the pathogenesis of peptic ulcers regardless of the presence of *H. pylori* infection [86] Related to the bacterial load [48] Involved in the activation of NF-κB → stimulates the proliferation of malignant gastric [50]	Decrease in the IL-1β level along with an increase in TNF-α might be considered a risk factor for peptic ulcer disease in the setting of *H. pylori* infection [86] Is associated with CRP and lower survival rates in patients with gastric carcinoma [87] → triggers a strong pro-inflammatory response in patients with *H. pylori* [86] IL-1β 511 T and IL-8 251 T gene polymorphisms → increase the virulence of *H. pylori* → increase the risk for gastric cancer [44] Upregulation and increased secretion of IL-1β → a major causative factor for hyperproliferation of gastric epithelial cells and subsequent oncogenesis [88]
**IL-18**	-	Role in gastric carcinogenesis [50] Promotes angiogenesis [50] Activates the NF-κB pathway, therefore interacting with other cytokines that have the same effect [50]	IL-18 serum levels → a prognostic factor for patients with gastric cancer [50] → patients with increased IL-18 serum levels present a lower survival rate [50]
**IL-10**	Encoded by a protein located on chromosome 1 [43]	Downregulates both cytotoxic inflammatory responses and cell-mediated immune responses [89] Associated with a higher risk for developing gastric carcinoma in *H.*-*pylori*-positive patients [90] IL-10 has suppressive effects on inflammation activation [92] Regulates the function of antigen-presenting cells and both the synthesis and expression of macrophages and the pro-inflammatory cytokines produced by T-cells [93] The level of IL-10 is directly dependent on the gastric cancer stage, therefore influencing the prognosis [50]	The immunosuppressive role of IL-10 is widely used by *H. pylori* [50]
**TNF-α**	An endotoxin-induced glycoprotein [94,95]	Has the ability to induce fever, apoptosis, inflammation and cachexia [94,95] Mediates the acute inflammatory responses to Gram-negative bacteria [96] Acts as both a recruitment and an activator factor for monocytes, macrophages and neutrophils, stimulating their migration to the infection site [97] An important role in the etiology of other human disorders, such as Alzheimer’s disease, major depression, non-gastric cancers or inflammatory bowel disease [86] Upregulates the nitric-oxide-dependent pathway responsible for the inhibition of DNA repair and promotion of DNA damage, and as an inductor of angiogenesis [50]	Is significantly higher in *H.*-*pylori*-positive patients without nodular gastritis [48] Is an indicator of the severity of tissue damage in patients with peptic ulcers [86] Impacts the NF-κB pathway [50] → a bridge between *H. pylori* infection and gastric carcinogenesis *H. pylori* secretes a protein that is meant to stimulate TNF-α production, namely, the TNF-α-inducing protein (Tipα), which will enable *H. pylori* to act as a tumor promoter [100]; this protein is a contributor of *H.*-*pylori*-induced gastric carcinogenesis
**TGF β**	Consists of activins, inhibins, growth differentiation factors, bone morphogenetic proteins, TGF β isoforms and glial-cell-derived factors [103]	A continuous inflammatory mediator [109] Promotes the adherence of *H. pylori* to the host’s cells and their subsequent colonization [110] → an indicator of the severity of *H.*-*pylori*-related non-metaplastic atrophic gastritis [111] Increased in patients with *H.*-*pylori*-positive gastritis and peptic ulcers as compared with *H.*-*pylori*-negative subjects [113] TGF β signaling pathways seem to be involved in gastric cancer development	Predictor of poor prognosis in patients with gastric cancer [114]

## 3. Conclusions

The *H.*-*pylori*-associated inflammation represents the cornerstone for all the extraintestinal complications triggered by this infection, but most importantly, it is closely related to the carcinogenesis process. Multiple cytokines are involved in the onset and persistence of these inflammatory responses and the identification of their precise role is vital in designing potential targeted therapies for decreasing the incidence of extraintestinal complications and gastric cancer during adulthood.
